# Real-time cardiovascular magnetic resonance T1 and extracellular volume fraction mapping for tissue characterisation in aortic stenosis

**DOI:** 10.1186/s12968-020-00632-0

**Published:** 2020-06-22

**Authors:** Sören J. Backhaus, Torben Lange, Bo Eric Beuthner, Rodi Topci, Xiaoqing Wang, Johannes T. Kowallick, Joachim Lotz, Tim Seidler, Karl Toischer, Elisabeth M. Zeisberg, Miriam Puls, Claudius Jacobshagen, Martin Uecker, Gerd Hasenfuß, Andreas Schuster

**Affiliations:** 1 University Medical Center Göttingen, Department of Cardiology and Pneumology, Georg-August University, Robert-Koch-Str. 40, 37099 Göttingen, Germany; 2 German Center for Cardiovascular Research (DZHK), Göttingen, Germany; 3 Department of Diagnostic and Interventional Radiology, University Medical Center Göttingen, Robert-Koch-Str. 40, 37075 Göttingen, Germany; 4 Cluster of Excellence “Multiscale Bioimaging: from Molecular Machines to Networks of Excitable Cells” (MBExC), University of Göttingen, Göttingen, Germany

**Keywords:** Real-time, T1 mapping, Aortic stenosis, Transfemoral aortic valve replacement, Tissue characterisation

## Abstract

**Background:**

Myocardial fibrosis is a major determinant of outcome in aortic stenosis (AS). Novel fast real-time (RT) cardiovascular magnetic resonance (CMR) mapping techniques allow comprehensive quantification of fibrosis but have not yet been compared against standard techniques and histology.

**Methods:**

Patients with severe AS underwent CMR before (*n* = 110) and left ventricular (LV) endomyocardial biopsy (*n* = 46) at transcatheter aortic valve replacement (TAVR). Midventricular short axis (SAX) native, post-contrast T1 and extracellular volume fraction (ECV) maps were generated using commercially available modified Look-Locker Inversion recovery (MOLLI) (native: 5(3)3, post-contrast: 4(1)3(1)2) and RT single-shot inversion recovery Fast Low-Angle Shot (FLASH) with radial undersampling. Focal late gadolinium enhancement was excluded from T1 and ECV regions of interest. ECV and LV mass were used to calculate LV matrix volumes. Variability and agreements were assessed between RT, MOLLI and histology using intraclass correlation coefficients, coefficients of variation and Bland Altman analyses.

**Results:**

RT and MOLLI derived ECV were similar for midventricular SAX slice coverage (26.2 vs. 26.5, *p* = 0.073) and septal region of interest (26.2 vs. 26.5, *p* = 0.216). MOLLI native T1 time was in median 20 ms longer compared to RT (*p* < 0.001). Agreement between RT and MOLLI was best for ECV (ICC > 0.91), excellent for post-contrast T1 times (ICC > 0.81) and good for native T1 times (ICC > 0.62). Diffuse collagen volume fraction by biopsies was in median 7.8%. ECV (RT *r* = 0.345, *p* = 0.039; MOLLI *r* = 0.40, *p* = 0.010) and LV matrix volumes (RT *r* = 0.45, *p* = 0.005; MOLLI *r* = 0.43, *p* = 0.007) were the only parameters associated with histology.

**Conclusions:**

RT mapping offers fast and sufficient ECV and LV matrix volume calculation in AS patients. ECV and LV matrix volume represent robust and universally comparable parameters with associations to histologically assessed fibrosis and may emerge as potential targets for clinical decision making.

## Introduction

Left ventricular (LV) remodelling including hypertrophy, interstitial volume alteration and expansion is a common feature in cardiac diseases [1]. Severe aortic stenosis (AS) is the most prevalent valvular heart disease in the Western world with 7 million people aged above 75 years thought to be affected [2, 3]. Severe symptomatic AS is associated with worse prognosis in the absence of aortic valve replacement [4–6]. However, prognosis after valve replacement may not entirely rely on improved flow-dynamics but depends on the level of collagen deposition during remodelling before intervention [7, 8]. Diffuse fibrosis precedes focal replacement fibrosis [9, 10], which has prognostic implications in asymptomatic AS patients [11]. Current guidelines recommend aortic valve replacement in symptomatic severe AS [12] irrespective of cardiac remodelling. Notwithstanding, there is evidence to suggest reverse myocardial remodelling following valve replacement with regression of diffuse fibrosis in some patients and consequently the quantification of its extent could represent a novel endpoint for patient selection [13]. Cardiovascular magnetic resonance (CMR) imaging allows quantification of myocardial remodelling using T1 mapping and extracellular volume (ECV) fraction calculation based on pre- and post-contrast T1 maps [10]. However, conventional Modified Look-Locker Inversion recovery (MOLLI) sequences [14], require retrospective acquisition over several heart beats and considerable breath holds which may be less accurate in dyspnoeic patients or for stress applications such as ischaemia assessment [15]. Recently, real-time (RT) T1 mapping based on inversion recovery Fast Low Angle Shot (FLASH) has been introduced which may overcome these limitations [16]. Whilst histology represents the reference standard, data comparing mapping and histology remains controversial [17, 18] and RT assessment has not yet been compared against collagen quantification based on endomyocardial biopsies. Hence, the aim of the present study was the evaluation of novel RT mapping sequences versus conventional MOLLI techniques and histological presence of fibrosis in AS [16, 19].

## Methods

### Study population

Between January 2016 and August 2019 patients who underwent transcatheter aortic valve replacement (TAVR) following heart team consensus decision were approached for study participation. Ethical approval was obtained from the local ethics committee. All patients gave written informed consent before participation. The study was conducted according to the principles of the Helsinki Declaration. The study was funded by a grant from the German Research Foundation (DFG, CRC 1002, D1).

### Cardiovascular magnetic resonance imaging

**Fig. 1:**
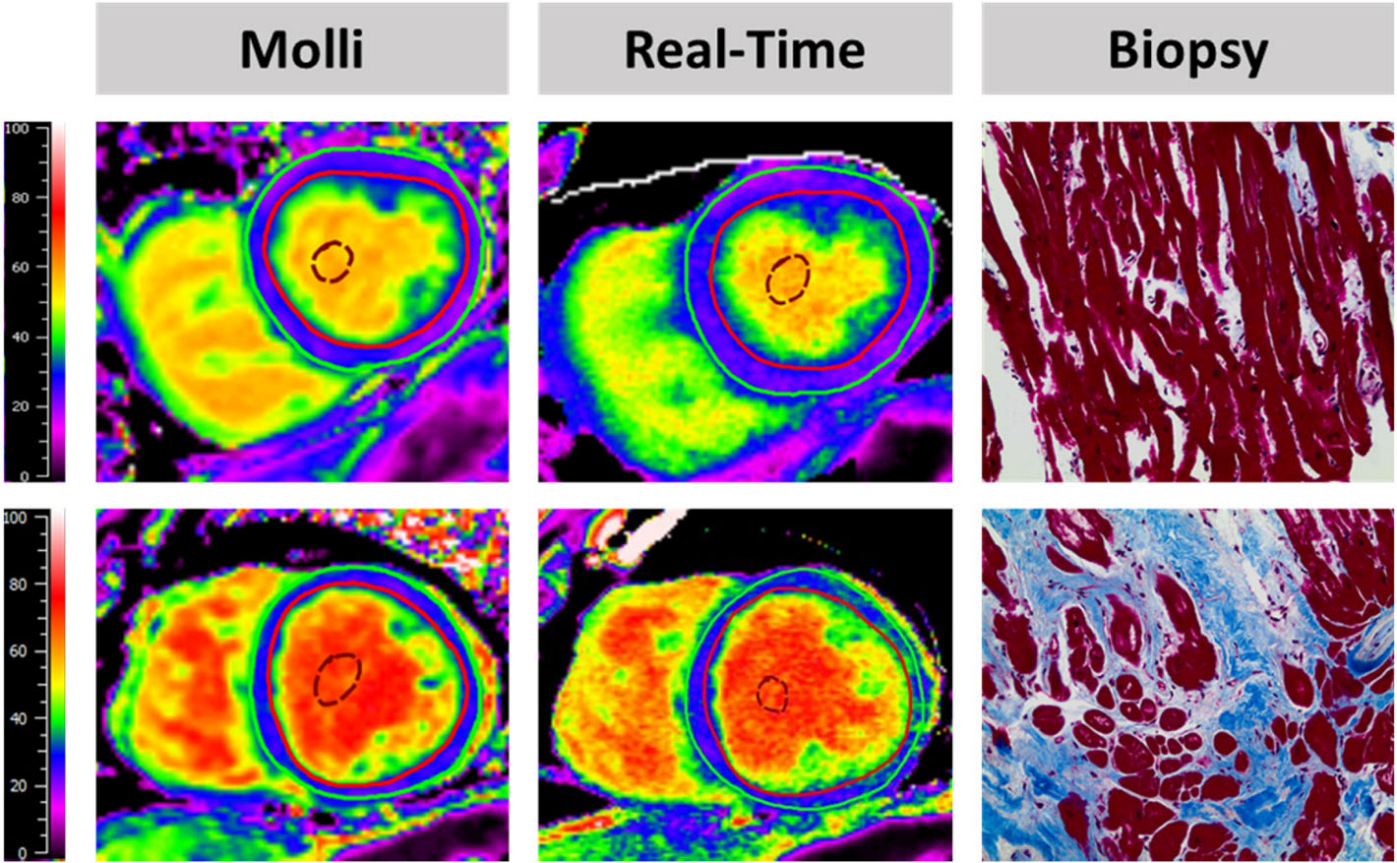
The upper half of the figure shows a histological slice of a biopsy from a patient with in total 4% fibrosis classified as perivascular and interstitial. The corresponding MOLLI (26.8%) and Real-time (26.6%) maps quantify extracellular volume fractions (ECV) within normal range. The lower half shows a histological slice of a biopsy containing a total of 21% fibrosis comprising 80% perivascular and interstitial fibrosis. The corresponding MOLLI (29.5%) and Real-time (30.1%) maps quantify ECV above average indicating pathology

CMR imaging was carried out using a standard protocol including balanced steady state free precession (bSSFP) functional imaging, native mapping prior to gadolinium application and post-contrast mapping 20 min after contrast administration as well as late gadolinium enhancement (LGE) imaging. Cardiac volumes were assessed in short axis (SAX) stacks acquired using electrocardiogram(ECG) gated bSSFP cine imaging. Mapping sequences comprised commercially available MOLLI (native: 5(3)3, post-contrast: 4(1)3(1)2) sequences [19–21] (Fig. 1) (MOLLI T1 mapping) with typical imaging parameters comprising a field-of-view (FOV) of 360 × 306.6 mm^2^, in-plane resolution 1.41 × 1.41 × 8 mm^3^, TR/TE = 2.7/1.12 ms, nominal flip angle 35°, bandwidth 1085 Hz/pixel and a total acquisition of 11 heart beats. Additionally, RT T1 single-shot myocardial T1 maps [16] (RT T1 mapping) of the mid-LV slices were acquired (Fig. 1) at a nominal in-plane resolution of 1.0 × 1.0 mm^2^ and 8 mm slice thickness using a FOV 256 × 256 mm^2^ in combination with a resolution of 512 complex data points per radial spoke (two-fold oversampling). Other parameters were TR/TE = 2.67/1.66 ms, nominal flip angle 4°, bandwidth 850 Hz/pixel and total acquisition time of 4 s following a non-selective adiabatic inversion pulse. Correct overlapping of underlying T1 weighted images was checked by the operator prior to drawing of the respective region of interest (ROI). The ROI was then propagated to all underlying T1 weighted images to create a midventricular SAX slice coverage and a septal ROI separately. To avoid partial volume effects of the blood pool or adjoining non-myocardial structures ROI delineation was carefully reviewed on all underlying T1 weighted images. ECV was calculated for the entire myocardium as well as the septal ROI separately as recently published [22]. To specifically evaluate diffuse processes, T1 and ECV ROIs excluded focal fibrosis/scars represented by focal LGE. Calculation of matrix volumes were performed using the product of LV myocardial volume (LV mass divided by specific gravity of myocardium [1.05 g/mL]) and ECV or (1 – ECV) for cellular volumes [13]. LGE imaging for the evaluation of ischemic scar was performed using inversion-recovery-gradient echo sequences 10–20 min after the administration of gadolinium-based contrast agents (0,15 mmol/kg). Post-processing analyses were performed using Medis (version 3.1.16.8, Medical Imaging Systems, Leiden, Netherlands). Quantifications were performed by observers blinded to clinical and histological or imaging characteristics respectively.

### Histological fibrosis assessment

LV endomyocardial biopsies were taken during TAVR procedures prior to replacement and from the anteroseptal basal myocardial segment using a Proflex-Bioptom (7 F, Medical Imaging Systems). The study procedure accounted for additional 5 to 10 min of intervention time. TAVR and CMR were performed within 48 h. Biopsies were fixed in 10% paraformaldehyde (PHA) and embedded in paraffin. Fibrosis was assessed using quantitative morphometry (cell-Sens 1.6, Olympus Corporation, Tokyo, Japan) and defined as blue area in Masson trichrome staining (MTS, section with positive staining for collagen) in relation to total tissue area. Collagen volume fraction (CVF) was defined as the amount of total fibrosis, including subendocardial fibrosis (ischemic), interstitial diffuse fibrosis (reactive) and replacement (scar) fibrosis [17] (Fig. 1), in relation to the total area of the biopsy. To precisely define the amount of interstitial myocardial fibrosis areas of subendocardial or replacement fibrosis were then excluded from the analysis volume in a second step with the final volume only including cardiomyocytes and interstitial fibrosis. Subsequently, diffuse CVF (dCVF) was defined as the percentage of interstitial fibrosis in relation to the remaining volume respectively.

### Statistics

Continuous parameters are presented as median values with interquartile range (IQR) after testing for normal distribution using the Shapiro-Wilk test. Non-normally distributed parameters underwent logarithmic transformation to achieve normal distribution. Dependent variables were compared using the Wilcoxon signed-rank test. Correlations were assessed using Pearson correlation coefficients. *P*-values provided are two-sided, an alpha level of 0.05 and below was considered statistically significant. Statistical agreement and variability were assessed in 20 randomly selected patients using the coefficient of variation (CoV, SD of mean difference divided by the mean) and intra-class correlation coefficients (ICC). Intra-observer reproducibility was performed with 4 weeks in between repeated analyses. ICCs were evaluated based on a model of absolute agreements as well as on a model for consistency [23]. ICC was considered excellent for values ≥0.74, good ≥0.60, fair ≥0.4 and poor < 0.40. Bland-Altman analyses [mean difference between measurements with 95% confidence interval (CI)] were performed to visualize agreements and variability [24]. Statistical analyses were calculated using SPSS (version 24, Statistical Package for the Social Sciences, International Business Machines, Inc., Armonk, New York, USA,) and Microsoft Excel (Microsoft, Redmond, Washington, USA).

## Results

### Study population

Baseline characteristics are reported in Table 1. In total 110 patients agreed for participation in the CMR study. LV endomyocardial biopsies were taken in 46 patients [25]. There was one biopsy induced papillary muscle injury resulting in an increase from moderate to severe mitral regurgitation which was successfully treated with Mitraclip (Abbott, Abbott Park, Illinois, USA) implantation leading to subsequent reduction to mild mitral regurgitation.

**Table 1:** Baseline characteristics

Parameter	Total
Sex (f/m)	63/42
Age	79 (75, 82)
BMI	27.7 (24.7, 30.8)
**Cardiovascular Risk Factors**	
Hypertension	93
Diabetes	37
Dyslipidaemia	64
Active smoking	8
Atrial fibrillation	16
**Cardiovascular magnetic resonance**	
LV EDVI (ml/m^2^)	79 (65, 98)
LV ESVI (ml/m^2^)	31 (18, 45)
LV EF (%)	60.5 (47.4, 72.0)
LV Mass (g/m^2^)	85 (69, 105)
LGE (g)	26.1 (14.7, 45.8)
**NYHA**	
I, II, III, IV	6, 35, 60, 4
**Laboratory Testing**	
NTproBNP (pg/ml)	1452 (608, 3916)
Creatinine (mg/dl)	1.03 (0.86, 1.19)
eGFR (ml/min/1.73m^2^)	60.0 (50.4, 75.3)
**Echocardiography**	
Peak aortic valve velocity (m/s)	4.0 (3.6, 4.6)
Mean Gradient (mmHg)	38 (29, 53)
Aortic valve area (cm^2^)	0.8 (0.6, 0.9)

Values are reported given as median and interquartile range or absolute numbers and associated percentage as appropriate.

*LV* left ventricular, *EDVI/ESVI* end-diastolic/systolic volume index, *EF* ejection fraction, *eGFR* estimated glomerular filtration rate; *LGE* late gadolinium enhancement; *NYHA* New York Heart Association; *BMI* body mass index

### Tissue characterisation

#### Histology

CVF as assessed by biopsy was in median 10.5% (IQR 3.0, 31.3) with 7% (IQR 2.5, 12.4) histologically defined as dCVF. Replacement fibrosis was present in 10 biopsies ranging between 1 and 76% of CVF within these biopsies. Subendocardial fibrosis was present 18 biopsies ranging between 1 and 81% of CVF of the biopsies. After accounting for different fibrosis pattern, dCVF accounted for 7.8% of total biopsy area (IQR 2.8, 14.3).

#### Imaging

**Fig. 2:**
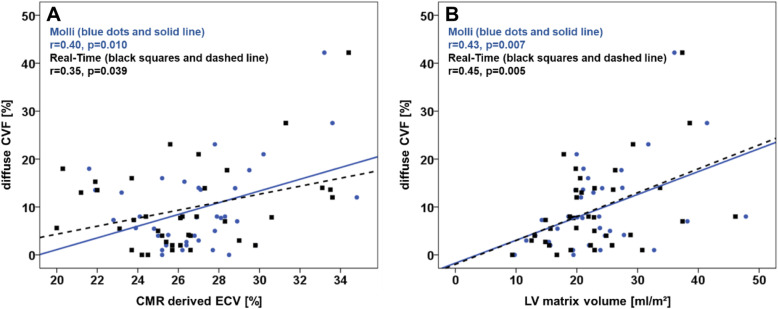
Correlation of diffuse collagen volume fraction (CVF) with cardiovascular magnetic resonance (CMR) derived (**a**) extracellular volume fraction (ECV) mapping and (**b**) left ventricular (LV) matrix volume using conventional (MOLLI) and Real-Time imaging

CMR derived tissue characterisation is reported in Table 2. Midventricular SAX slice coverage as well as septal ROI T1 times as assessed by MOLLI sequences were significantly longer compared to RT assessments both prior to and after contrast application (*p* < 0.001 for all). However, calculated ECV were similar for midventricular SAX slice coverage (*p* = 0.073) as well as the septal ROI (*p* = 0.216). Differences between MOLLI and RT sequences were numerically smaller for the septal ROI compared to the midventricular SAX slice coverage. ECV (MOLLI *r* = 0.40, *p* = 0.010; RT *r* = 0.35, *p* = 0.039, Fig. 2) and ECV derived LV matrix volume (MOLLI r = 0.43, *p* = 0.007; RT *r* = 0.45, *p* = 0.005) were the only parameter associated with dCVF as assessed by biopsies. In contrast, ECV derived cellular volume did not correlate significantly with dCVF (MOLLI *r* = 0.29, *p* = 0.081; RT *r* = 0.28, *p* = 0.100). There was no correlation comparing the proportion of dCVF in relation to healthy myocardium to native T1 (5(3)3 MOLLI *r* = 0.08, *p* = 0.600; RT *r* = 0.09, *p* = 0.547) or post-contrast T1 (MOLLI *r* = 0.02, *p* = 0.883; RT *r* = 0.01, *p* = 0.933). Furthermore, there was no correlation between histologically assessed total fibrosis and neither 5(3)3 MOLLI derived native T1 (*r* = − 0.23, *p* = 0.143) or ECV (*r* = 0.21, *p* = 0.197) nor RT native T1 mapping (*r* = 0.15, *p* = 0.356) or ECV (*r* = 0.21, *p* = 0.211) assessments. Out of 16 patients with atrial fibrillation, 4 patients had combined data on CMR and histologically derived fibrosis. Correlation coefficients of ECV and dCVF were numerically higher for RT (*r* = 0.60, *p* = 0.40) as opposed to MOLLI (*r* = 0.36, *p* = 0.64).

**Table 2:** Left ventricular tissue characterisation

	MOLLI Midventricular SAX	Real-time Midventricular SAX	p-value	MOLLI Septal ROI	Real-time Septal ROI	*p*-value
Native	1309 (1274, 1345)	1289 (1248, 1313)	< 0.001	1310 (1287, 1342)	1291 (1261, 1322)	< 0.001
Post-contrast	520 (490, 550)	487 (451, 518)	< 0.001	525 (489, 554)	504 (470, 533)	< 0.001
ECV	26.5 (24.9, 27.8)	26.2 (24.4, 27.7)	0.073	26.5 (24.2, 28.2)	26.2 (24.4, 27.7)	0.216
Matrix volume (ml/m^2^)	21.0 (17.9, 26.3)	20.3 (17.0, 26.9)	0.059			
Cell volume (ml/m^2^)	59.0 (47.8, 74.2)	59.2 (47.3, 73.5)	0.059			

T1 values are reported as median in milliseconds with associated interquartile range. *P*-values were calculated using the Wilcoxon signed-rank test. *ECV* extracellular volume in %. SAX, short axis; *ROI* region of interest

### Agreement of fibrosis assessments

**Fig. 3:**
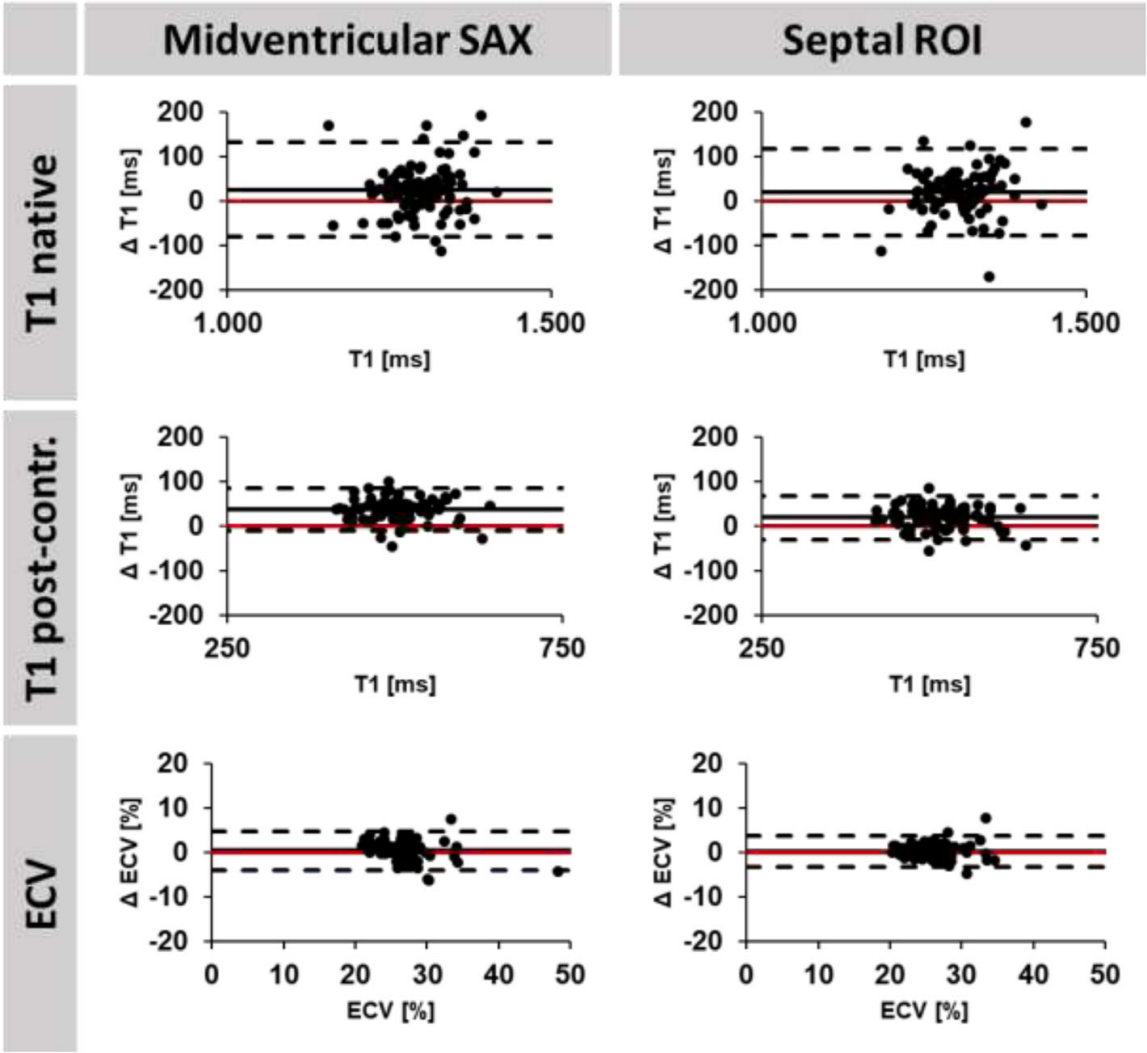
Bland Altman plots are shown for native T1, post-contrast T1 and extracellular volume fraction (ECV) comparing MOLLI and Real-time based acquisitions for midventricular short axis (SAX) and septal regions of interest (ROI). Δ: difference (MOLLI - Real-Time). The red line indicates 0 difference

**Fig. 4:**
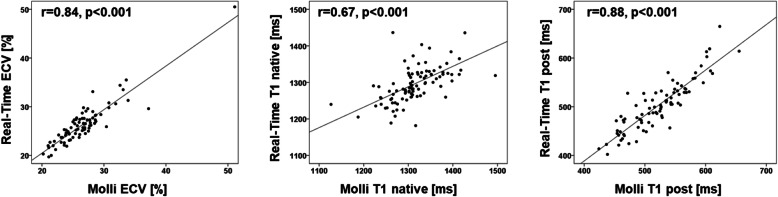
Correlation is shown for Real-time and MOLLI derived septal extracellular volume fraction (ECV), native and post-contrast T1 times

Agreements of MOLLI and RT mapping are reported in Tables 3 and 4 as well as Figs. 3 and 4. Statistical agreements comparing assessments by MOLLI and RT sequences are higher for the septum compared to the myocardium. This is also reflected by higher correlation coefficients for septal assessments. Statistical agreements are good for native T1 times (ICC > 0.62), excellent for post-contrast T1 times (ICC > 0.81) as well as excellent and best for ECV (ICC > 0.91) irrespective of midventricular septal or midventricular SAX slice coverage. Intra- and interobserver reproducibility of T1 times and ECV were consistently excellent (Table 5).

**Table 3:** Reproducibility Mapping

Method	Parameter	Mean Difference (SD of the Diff.)	ICC (95% CI)	CoV (%)
MOLLI vs. Real-time	T1 midventricular SAX native	25.4 (53.0)	0.62 (0.38–0.769	4.1%
	T1 septal ROI native	19.7 (48.8)	0.69 (0.50–0.80)	3.7%
	T1 midventricular SAX post-contrast	37.3 (23.9)	0.81 (0.00–0.94)	4.7%
	T1 septal ROI post-contrast	19.1 (24.2)	0.91 (0.65–0.96)	4.7%
	ECV midventricular SAX	0.31 (2.23)	0.91 (0.85–0.94)	8.3%
	ECV septal ROI	0.24 (1.75)	0.95 (0.93–0.97)	6.6%

The ICC was calculated for absolute agreement of the strain values. *SD* standard deviation; *Diff* Difference; *ICC* intraclass correlation coefficient; *CoV* coefficient of variation; *ECV* extracellular volume; *ROI* region of interest. Native: prior to Gadolinium contrast agent application, post: 15 min post application

**Table 4:** Correlation MOLLI vs. Real-time Mapping

Method	Parameter	correlation	p
MOLLI vs. Real-time	T1 midventricular SAX native	0.56	< 0.001
	T1 septal ROI native	0.66	< 0.001
	T1 midventricular SAX post-contrast	0.86	< 0.001
	T1 septal ROI post-contrast	0.88	< 0.001
	ECV midventricular SAX	0.73	< 0.001
	ECV septal ROI	0.84	< 0.001

*ECV* extracellular volume; *ROI* region of interest

**Table 5:** Intra- and Inter-observer reproducibility

Observer	Parameter	Mean Difference (SD of the Diff.)	ICC (95% CI)	CoV (%)
Intra-observer	T1 midventricular SAX native	6 (7)	0.99 (0.96–1.00)	0.6%
MOLLI	T1 septal ROI native	2 (11)	0.99 (0.97–1.00)	0.9%
	T1 midventricular SAX post-contrast	8 (15)	0.97 (0.89–0.99)	2.9%
	T1 septal ROI post-contrast	4 (14)	0.98 (0.94–0.99)	2.8%
	ECV midventricular SAX	0.44 (0.76)	0.97 (0.91–0.99)	2.9%
	ECV septal ROI	0.02 (0.60)	0.99 (0.97–1.00)	2.4%
Intra-observer	T1 midventricular SAX native	6 (5)	0.99 (0.91–1.00)	0.4%
Real-time	T1 septal ROI native	1 (8)	1.00 (0.99–1.00)	0.6%
	T1 midventricular SAX post-contrast	5 (4)	1.00 (0.94–1.00)	0.9%
	T1 septal ROI post-contrast	2 (5)	1.00 (0.99–1.00)	1.1%
	ECV midventricular SAX	0.42 (0.45)	0.98 (0.88–1.00)	1.7%
	ECV septal ROI	0.10 (0.62)	0.99 (0.97–1.00)	2.4%
Inter-observer	T1 midventricular SAX native	7 (14)	0.98 (0.94–0.99)	1.1%
MOLLI	T1 septal ROI native	1 (15)	0.98 (0.94–0.99)	1.2%
	T1 midventricular SAX post-contrast	3 (4)	1.00 (0.98–1.00)	0.8%
	T1 septal ROI post-contrast	1 (5)	1.00 (0.99–1.00)	1.0%
	ECV midventricular SAX	0.35 (0.76)	0.98 (0.94–0.99)	3.0%
	ECV septal ROI	0.09 (0.45)	0.99 (0.99–1.00)	1.8%
Inter-observer	T1 midventricular SAX native	7 (9)	0.99 (0.95–1.00)	0.7%
Real-time	T1 septal ROI native	3 (11)	0.99 (0.99–1.00)	0.8%
	T1 midventricular SAX post-contrast	4 (5)	1.00 (0.97–1.00)	1.0%
	T1 septal ROI post-contrast	1 (6)	1.00 (0.99–1.00)	1.3%
	ECV midventricular SAX	0.34 (0.48)	0.99 (0.95–1.00)	1.8%
	ECV septal ROI	0.04 (0.36)	1.00 (0.99–1.00)	1.4%

*SD* standard deviation; *ICC* intraclass correlation coefficient; *CoV* coefficient of variation; *ECV* extracellular volume; *ROI* region of interest; *SAX* short axis

## Discussion

The present study reports 4 major findings. First, novel RT native T1 and ECV mapping agrees well with MOLLI sequences suggesting adequate estimation of myocardial fibrosis. Second, the relationship between fibrosis assessment by CMR and LV endomyocardial biopsy derived histology is complex and both modalities provide different and complementary information. Third, ECV and LV matrix volume are the only parameters showing a significant correlation with dCVF and may therefore serve as non-invasively obtained surrogates of biopsy derived histological extent of interstitial fibrosis with potential implications for clinical decision making and follow up surveys. Last, RT mapping may be particularly suited for examinations depending on high temporal resolution e.g. in patients prone to dyspnoea, arrhythmias or stress mapping.

The present data demonstrates good agreement between novel RT and 5(3)3 MOLLI mapping techniques for native T1 values, which is generally better for the septum as compared to the overall myocardium. Higher and excellent agreement is found for ECV for midventricular SAX slice coverage and the septal ROI, showing no statistical numerical difference between RT and MOLLI sequences. Additionally, and in opposite to native or post-contrast T1, ECV is less prone to factors influencing magnetization thus being more universally comparable [26]. The potential clinical value for ECV is further underlined by robust intra- and inter-observer reproducibility and recent data showing ECV to be more strongly associated with outcome compared to native T1 in over 1700 patients with myocardial disease [27].

In contrast, agreement with fibrosis assessment by histology is low. Neither native 5(3)3 MOLLI nor RT T1 as well as ECV mapping did correlate with total fibrosis, which is a distinct deviation from previous assumptions [18, 28] but in line with recent data presented by Treibel et al. [17]. It is important to remember that both methods (histology and CMR based) are different with inherent advantages and disadvantages.

First, despite of limited guidance for biopsies performed in the cath-lab and their focal nature of assessment of endomyocardial tissue, biopsies are considered to represent the whole ventricular myocardium. Biopsies performed in the CMR suite may overcome limitations owed to guidance [29], nevertheless the major concern of their focal nature remains. In contrast, CMR assessments comprise myocardial changes over the LV in its entirety. Furthermore, biopsies offer in depth tissue characterisation on the microscopic, molecular, immunological and genetic level allowing deep and comprehensive assessments but carry the risk of the procedure. CMR offers non-invasive radiation-free tissue characterisation using native and/or contrast-agent based approaches for diffuse and focal tissue characterisation [1]. However limits of CMR include spatial resolution [16] and the dependency of magnetic fields and different mapping protocols leading to hard- and software specific native T1 times and differing normal values. Whilst ECV and LGE are more universally comparable, they are dependent on the use of gadolinium based contrast-agents which are relatively contraindicated in patients with severe renal failure [30]. Biopsies however, may be performed in this subgroup as well.

Second, it has to be noted that ECV reflects diffuse myocardial fibrosis. Reports for the correlation of ECV to histologically assessed fibrosis vary. Better correlation than ours was found by de Meester de Ravenstein [31] reporting good correlation of ECV and histologic fibrosis (*r* = 0.78) using a 3-(3)-3-(3)-5 MOLLI sequence. Multiple factors may contribute to this difference. It could be partially related to the different sequence used, it could be influenced by lower sample size and time differences between CMR and biopsy (1 to 30 days) or the size of the latter, since patients undergoing open-chest aortic surgery underwent biopsies of the full width of myocardium as opposed to our endomyocardial biopsy. In line with our data de Meester de Ravenstein also reported low correlation for native T1 to histology. Kammerlander et al. [32] reported moderate correlation of ECV and histology (*r* = 0.49) but association to cardiovascular outcome in 473 consecutive patients referred to CMR. Similar to Treibel et al. [17] we did not observe a significant correlation of ECV and total CVF. Arguably, this may not entirely be surprising since interstitial myocardial fibrosis cannot be assessed in areas with subendocardial and focal replacement fibrosis. We therefore excluded these areas to precisely define the histological reference volume. Indeed, significant correlation was then established for ECV (diffuse fibrosis as assessed by CMR after exclusion of focal LGE) compared to dCVF (diffuse fibrosis in histology after exclusion of non-interstitial collagen deposition) calculated within the biopsies. Both MOLLI and RT ECV quantifications showed this association. Bearing in mind the value of diffuse myocardial remodelling and its potentially reversible nature preceding irreversible scaring, ECV may allow better patient selection for further treatment. However, whilst ECV is influenced by changes in cellular and interstitial volumes, calculation of LV matrix volumes might overcome this limitation [13]. Indeed, correlations with histological dCVF were highest for both RT and MOLLI derived LV matrix volumes.

Third, despite the focal nature of biopsies, one would expect lower but significant correlations of fibrosis and T1 as previously reported [18, 28]. The absence of statistical correlation may be based on methodological differences. T1 mapping may rather reflect the entirety of myocardial changes including fibrosis, inflammation and myocardial hypertrophy [1] as opposed to strict fibrosis assessment by staining in histology. Puls et al. [33] have recently underlined the impact of fibrosis on the prognosis of patients following TAVR. Everett et al. [21] demonstrated that ECV but not native T1 assessment yields prognostic value in patients with AS undergoing TAVR. This is in line with the present observation of correlations between ECV and matrix volume but not native T1 mapping with invasively derived fibrosis quantification. Non-invasive tissue characterisation using ECV or matrix volume quantification may therefore offer important non-invasive clinical information in addition to currently established diagnostic and therapeutic pathways [12].

Technical challenges in MOLLI T1 mapping comprise banding artefacts depending on magnetic field strengths. CMR imaging in general is prone to artefacts caused by inadequate breath-holding. MOLLI sequences require a breath hold for up to 17 heartbeats [16]. Consequently, shorter mapping protocols were demonstrated to be better tolerated [34]. CMR RT imaging [35] aims for an approach capable of cardiac imaging with limited or without use of breath-holding. RT mapping, using single-shot inversion recovery FLASH sequences for high spatial and temporal resolution reduces the extent of breath-holding to 4 seconds [16]. This offers unique opportunities for CMR imaging in patient collectives more prone to dyspnoea such as AS or congested patients. Another area of applications includes stress testing. Adenosine stress mapping has proven incremental value for the detection of relevant coronary artery disease [15] without the need for contrast application. Furthermore, free breathing T1 mapping has shown feasibility during exercise testing [36]. Indeed, exercise stress in combination with native mapping could potentially eliminate drug application [37]. No contrast application is of major advantage for patients with renal failure referred for coronary artery disease assessment. Furthermore, the value of conventional CMR is limited in patients with arrhythmia e.g. caused by atrial fibrillation due to the ECG gated retrospective alignment of cardiac phases over several heartbeats. RT imaging overcomes these technical limitations [35]. In our study population in AF patients, agreement of RT ECV and histologically derived dCVF was numerically higher compared to MOLLI derived ECV and dCVF. However, with only 4 participants in AF and complete CMR and histological data, this can only be considered hypothesis generating. These advances in the field of fast mapping hold promising value for future clinical applications offering fast, detailed and reliable tissue characterisation by RT mapping. Despite advances made, clinical studies are warranted to investigate the value and reliability of novel mapping for hard clinical endpoints.

Furthermore, none of the above covers the entire pathophysiology of AS [17]. Whilst correlation between CMR and histology was statistically significant, it remained low (r ≤ 0.4 for ECV vs dCVF). Limited agreements may however also point towards beneficial complimentary use of both strategies, which should be defined in future prospective research studies.

### Study limitations

Whilst intra- and inter-observer reproducibility was assessed underlining excellent post-processing reproducibility, data on inter-study reproducibility is lacking which would be desirable for follow-up surveys of cardiac remodelling. With this perspective, further clinical follow-up studies are warranted. Mapping was performed in one representative midventricular SAX slice only, future studies should consider an apical, midventricular and basal slice for more comprehensive assessments. We have not demonstrated a significant association of native and post contrast T1 mapping based on RT and MOLLI (native 5(3)3 and post-contrast 4(1)3(1)2) with histology. This may partially be due to the specific sequences used. Both T1 mapping sequences utilized are affected by imperfect inversion due to T2 relaxation. While the MOLLI sequence is additionally affected by T2 and magnetization transfer effects during the bSSFP readout (Robson et al. [38]), this does not affect the RT single-shot T1 mapping sequence which uses a FLASH readout as in the original approach by Deichmann and Haase [39]. However notwithstanding it is important to understand that native and postcontrast T1 based on various T1 mapping techniques did not correlate with outcome in a recent AS multi-center study while ECV did predict outcome in these patients [21]. Furthermore, ECV did correlate with histological fibrosis in the current study, which itself was demonstrated to have strong prognostic implications [33]. Last, only a subset of patients underwent endomyocardial biopsies which limits statistical power.

## Conclusion

RT mapping offers fast and sufficient ECV and LV matrix volume calculation in AS patients. RT ECV and LV matrix volume represent robust and universally comparable parameters that agree well with those derived from a specific MOLLI sequence. Since they show associations to histologically assessed fibrosis they may well emerge as a potential target for clinical decision making.

## Data Availability

Regarding data availability, we confirm that all relevant data are within the paper and all data underlying the findings are fully available without restriction and can be accessed at the University Medical Centre Goettingen by researchers who meet the criteria for access to confidential data.

## Abbreviations

AF: Atrial fibrillation
AS: Aortic stenosis
BMI: Body mass index
CI: Confidence interval
CMR: Cardiovascular magnetic resonance
CoV: Coefficient of variation
CVF: Collagen volume fraction
dCVF: Diffuse collagen volume fraction
ECG: Electrocardiogram
ECV: Extracellular volume fraction
EDVI: End-diastolic volume index
EF: Ejection fraction
ESVI: End-systolic volume index
eGFR: Estimated glomerular filtration rate
FLASH: Fast low angle shot
FOV: Field of view
HFpEF: Heart failure with preserved ejection fraction
ICC: Intra-class correlation coefficients
LGE: Late gadolinium enhancement
LV: Left ventricle/left ventricular
MOLLI: Modified look-locker inversion recovery
NYHA: New York Heart Association
ROI: Region of interest
RT: Real-Time
SAX: Short axis
TAVR: Transcutaneous aortic valve replacement
